# Application of Digital Molding in Maxillofacial Prosthetics: A Narrative Review: Application of Digital Molding in Maxillofacial Prosthetics

**DOI:** 10.31661/gmj.v13iSP1.3656

**Published:** 2024-12-08

**Authors:** Maryam Jahangiri, Seyed Mohammad Reza Hakimaneh, Mohammad Amin Bafandeh, Fatemeh Bakhtiari, Sayed Shojaedin Shayegh

**Affiliations:** ^1^ Department of Prosthodontics, Faculty of Dentistry, Shahed University, Tehran, Iran

**Keywords:** 3D Imaging Tools, CT Scans, Maxillofacial Prosthesis

## Abstract

**Background:**

The use of digital tools and 3D molding has become very common in dentistry today. However, there are few studies on the possibility of using 3D imaging tools for molding maxillofacial defects. In this review study, we examine articles that have used digital molding tools instead of conventional methods for molding maxillofacial defects.

**Materials and Methods:**

In this study, all articles related to keywords of “3D imaging tools”, “CT scans”, “Maxillofacial Prosthesis” were collected and reviewed by searching PubMed and ISI Web of Science until 2024. Then, the materials were classified into the following topics: the use of intraoral scanners in molding for maxillofacial prostheses, the use of facial scanners in molding for maxillofacial prostheses, the use of CT scans in molding for maxillofacial prostheses, and the use of new digital methods in molding for ocular, nasal, ear prostheses, maxillary and mandibular obturators, soft palate defects, and nasoalveolar molding prostheses, and were examined in detail.

**Results:**

This study showed that depending on the type of defect, specific types of digital molding tools can be used to the greatest advantage. Intraoral scanners can be used in the construction of nasoalveolar moldings, obturators, cleft palate, and ear prostheses. Facial scanners have the highest accuracy for molding defects in the middle third of the face. Facial scanners are helpful in midface defects, and in the construction of ocular and nasal prostheses. The main use of CBCT molding is in molding the patient’s palate for the design and construction of obturators. For mandibular molding, the use of intraoral scanners is much better than other methods. Moreover, even in cases where the patient has mild to moderate trismus after mandibulectomy, the use of intraoral scanners has acceptable accuracy.

## Introduction

Maxillofacial defects can be caused by hereditary, acquired, or developmental factors [1]. The main way to reconstruct defects is through surgery, but surgical reconstruction of some of these defects is time-consuming or may not be possible. Maxillofacial prostheses are an integral part of maxillofacial treatments [1][2]. Maxillofacial prostheses can improve the quality of life of patients by restoring their appearance and function [3]. In 1953, Ackerman first introduced maxillofacial prostheses as a sub-branch of dentistry [1]. Maxillofacial prostheses are classified according to the area being reconstructed, into types of nasal, ear, eye, mandibular, maxillary obturator, molding nasoalveolar prostheses (to correct problems caused by cleft palate and lip), and prostheses that correct soft palate defects [4][5].

The main issue in making a good maxillofacial prosthesis is an accurate impression of the defects [6]. Maxillofacial prosthesis impression making is often difficult and time-consuming and is bothersome for the patient and difficult for the clinician [7]. In the mid-20th century, digitalization rapidly expanded in all industries and also encompassed the health system [6]. The use of these digital tools has made the impression-making process easier and faster for maxillofacial patients. Replacing conventional impression-making methods and landmark recording with the use of three-dimensional imaging is the future of dentistry. Some of the new impression-making methods in these patients include intraoral scanners, facial scanners, and CT scans [8][9][10].

The first method of producing a three-dimensional image is Cone Beam Computed Tomography (CBCT). Computed tomography (CT) was introduced in 1973 to create three-dimensional images. Years later, Cone Beam Computed Tomography (CBCT) was introduced for use in more limited fields such as the head and neck [11]. In CBCT, unlike CT, the X-ray beam is directed in a conical shape towards the desired area. This reduces the field of irradiation and reduces the patient’s exposure [12]. The applications of this imaging in the field of prosthetics include: implant prosthetics, imaging of the temporomandibular joint, maxillofacial prosthetics, diagnosis and evaluation of craniofacial problems, and finally, evaluation and causation of airway problems [13]. The main use of CBCT is in the construction of obturators after maxillectomy for oncological reasons [13][14].

One of the new methods is intraoral scanners. Dr. Francois Duret was one of the pioneers of using optical impressions, which he used in France in 1971. The first intraoral scanner was designed in Switzerland in 1980 by Professor Mormann. This scanner was the first generation of scanners [6]. Using intraoral scanners has many advantages and disadvantages. Intraoral scanners offer advantages like easy correction, digital file storage, no need for physical archiving, no impression materials, cost-effectiveness, infection prevention, improved communication with labs, enhanced patient satisfaction, and utility for patients with maxillofacial defects. The convenience, speed, and patient satisfaction associated with intraoral scanners have led to increased adoption among dentists [15].

The third method in digital impression of maxillofacial prostheses is the use of 3D facial scanners, which have been used since 1991. Moss and his colleagues examined the growth of children with deformities using laser scans [16]. The main use of 3D facial scanners is in smile design, orthodontic diagnoses such as asymmetry, and also recording before orthognathic and maxillofacial surgeries [17]. The improved accuracy of these tools has enabled their application in maxillofacial prosthesis impressions, although their use remains limited due to insufficient studies. Recent articles discuss the use of facial scanners for molding maxillofacial defects [18][19][20][21]. The maxillofacial defects may make the impression process difficult, time-consuming and annoying for the patient. Using digital tools can be very useful. Using these tools can make impression faster, easier and facilitate the work for the therapist and the patient. Digital impression in maxillofacial prostheses is a novel topic. Digital impression methods in maxillofacial patients is more important and helpful than other fields of dentistry, due to the time-saving, less annoying and more accurate. The studies conducted were not focused on the digital impression of maxillofacial defects. In this study, we put all the techniques and methods together in a practical way so that it is practical and useful for clinicians. In this review study, we are trying to review the new tools for the maxillofacial prostheses impression. For this purpose, we will classify and review the articles based on the construction of maxillofacial prostheses and also types of digital tools.

## Methods and Materials

In this study, we have searched in PubMed and ISI Web of Science with keywords of "3D imaging tools", "CT scans", "Maxillofacial Prosthesis" until 2024 and all related articles were collected and reviewed. Then, the outcomes were classified into the following topics: the use of intraoral scanners in molding for maxillofacial prostheses, the use of facial scanners in molding for maxillofacial prostheses, the use of CT scans in molding for maxillofacial prostheses, and the use of new digital methods in molding for ocular, nasal, ear prostheses, maxillary and mandibular obturators, soft palate defects, and nasoalveolar molding prostheses, and were reviewed in detail.

## Results and Discussion

**Figure-1:**
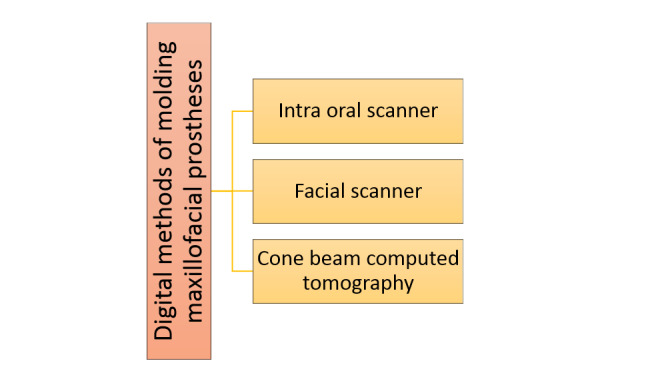
Figure 1. Classification of digital impression based on the methods

**Figure-2:**
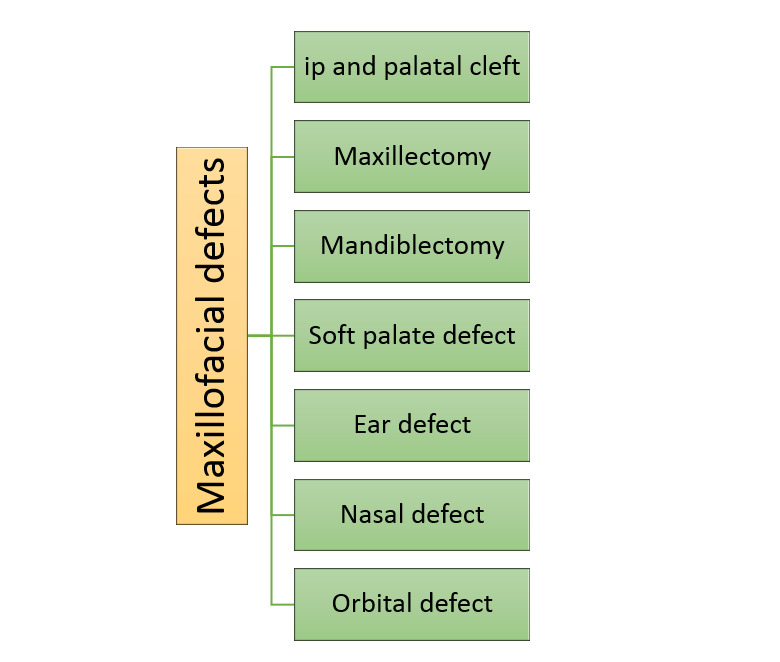
Figure 2. Maxillofacial defects that are examined in this study and their digital impression methods.

**Table:** Table1. Common Facial Scanners Used in Dentistry [38]

**Mechanism**	**Scanner**
**Photogrammetry** **/Stereophotogrammetry**	Planmeca ProMax 3D Mid(PM) (Planmeca USA, Inc.,Hoffman Estates, IL, USA)
	3dMD Face system (3dMD,Atlanta, GA, USA)
	Facehunter (Zirconzahn,South Tyrol, Italy)
	FaceScan system (Isravision,Darmstadt, Germany)
**Structured light scanner**	Priti mirror scanner and priti image software (Isravision,Polymetric, Germany)
	(Artec Space Spider; Artec 3D)
**Laser scanner**	ObiScanner (ObiScanner,Milano, Italy)
	NeXT Engine
**Mechanism**	Scanner
**Photogrammetry** **/Stereophotogrammetry**	Planmeca ProMax 3D Mid(PM) (Planmeca USA, Inc.,Hoffman Estates, IL, USA)
	3dMD Face system (3dMD,Atlanta, GA, USA)
	Facehunter (Zirconzahn,South Tyrol, Italy)
	FaceScan system (Isravision,Darmstadt, Germany)
**Structured light scanner**	Priti mirror scanner and priti image software (Isravision,Polymetric, Germany)
	(Artec Space Spider; Artec 3D)
**Laser scanner**	ObiScanner (ObiScanner,Milano, Italy)
	NeXT Engine

### 1. Classification of Digital Molding Based on the Methods

In this study, several methods for digital molding of maxillofacial lesions are examined. These methods are summarized in Figure-1.

### 1. 1. Intraoral Scanner

Generally, an intraoral scanner consists of three main components: an intraoral camera, a computer, and software. The process of creating a 3D image is performed by emitting light to the object, receiving the reflected light, and then determining the distance and forming an image. The mechanism for determining the distance of intraoral scanners is classified into different methods such as Confocal, Stereophotogrammetry, Active Wavefront Sampling, and Triangulation [22]. The use of intraoral scanners has several advantages and disadvantages. Some of the advantages include the ability to easily modify or rescan, to see the area and evaluate it during scanning, the ability to save the file digitally and not need to archive physical casts, the lack of use of impression materials, being cost-beneficial, not transmitting infection to the laboratory, easy communication with the laboratory, increased patient satisfaction, the possibility of scanning in patients who are unable to mold due to the gag reflex, and the ability to select color digitally [15].

The use of intraoral scanners in routine dentistry is common due to high speed, accuracy, patient comfort [23], the use of intraoral scanners in patients with maxillofacial defects has been considered. In the following, we will examine the studies that use intraoral scanners in maxillofacial defects.

1. Unkovskiy et al. in 2022 examined the accuracy of digital molding for orbital, nasal, and ear prostheses. In this study, two intraoral scanners, Trios 4 and Primescan, a facial scanner Pritiface, and a portable facial scanner Artec Space Spider, and a mobile phone were used. In this study, the accuracy of the mobile phone was low in all defects. The intraoral scanner Primescan had acceptable accuracy in nasal and ear defects, while both intraoral scanners were not recommended for ocular defects due to low accuracy [24].

2. Jacob et al. assessed the accuracy of two intraoral scanners, ITero™ and Lythos, and one extraoral scanner [Ortho Insight 3D™], in digital mandibular molding. This study showed clinically acceptable accuracy of intraoral scanners [25].

3. Patel et al. [26], ElNaghy et al. [27], and Okazaki et al. [28] suggested the use of intraoral scanners for palatal cleft.

4. Gong et al. [29] and Villarreal-Martínez et al. [30] suggested using intraoral scanners in the construction of Nasoalveolar Molding.

5. Brucoli et al. [8], Islam et al. [31], and Ye et al. [32] suggested using intraoral scanners in the construction of maxillary obturators.

6. Gao et al. recommend digital molding in mandibulectomy in conditions of mild and moderate trismus. In this study, only in severe trismus conditions, the use of intraoral scanners does not have sufficient accuracy [33].

7. Gadallah in 2023 suggested the use of intraoral scanners for molding of ear prostheses [34].

### 1. 2. Facial Scanner

Today, intraoral scanners have found their place in dental treatments and are a suitable replacement for the conventional method. The next step in digital molding is the use of 3D facial scanners, which are rapidly growing. 3D facial scanners generally have four operating mechanisms: laser-scan, photogrammetry, Structured light, and Stereophotography [35]. Stereophotography is a non-contact 3D imaging method of soft facial tissue. In this method, the person’s face is placed at an equal distance from cameras that take images simultaneously. In this way, an image is taken from different angles at one time, which, when superimposed on each other, produces a 3D image [36]. Structured light is a cost-effective method in which light is emitted from one side of the face and information is collected from the other side using a detector [37]. Photogrammetry is the creation of a 3D image from 2D using software [37]. In the laser-scan method, which is more accurate and expensive than other methods, a laser is directed at the face and the reflected rays are collected by a detector [37]. According to the study by Lee, a summary of the brands of facial scanners used in dentistry is compiled in Table-1 [38]. D’Ettorre et al. compared the accuracy of two methods: Structured light and Stereophotography. In this study, they used the 3dMDtrio facial 3d scanner (3dMD, Atlanta, Ga) and, for Structured light, the Bellus3D Face Application (version 1.6.11; Bellus3D Inc, Campbell, Calif/ a smartphone application facial scanner). This study introduced Stereophotography as the standard for facial scanning due to its high accuracy and speed. However, using a smartphone can be acceptable if enough time is taken and the operator is careful. The biggest advantage of using structured illumination is its portability and affordability [39].

Knoops et al. compared four 3D facial scanners: 1.5T Avanto MRI, 3dMDface System, M4D Scan, and Structure Sensor. This study identified the 3dMDface System and M4D Scan as the most accurate facial scanners [40].

The primary use of 3D facial scanners is in smile design, orthodontic diagnoses such as asymmetry, and recording before orthognathic and maxillofacial surgeries. [17] However, the increased accuracy of these digital tools has enabled their use for maxillofacial prosthesis molding. Although not widely used due to limited studies, the following studies have used facial scanners to create facial prostheses.

1. Zhao et al. assessed the accuracy of facial scanners in patients with facial deformities. In this study, the accuracy of two facial scanners, FaceScan system (structured illumination) and 3dMD Face system (Stereophotography), was compared to an industrial scanner, which is significantly more accurate than facial scanners. Scans were taken from 10 patients with maxillofacial problems. For each patient, the scan files were aligned and examined using the Geomagic software. This study examined accuracy in three areas: the upper, middle, and lower thirds of the face. This study showed the best accuracy of facial scanners in defects in the midface and was clinically acceptable. No difference was reported between the two facial scanner models [35].

2. In 2024, Park et al. designed a fully digital implant-based overdenture with a pharyngeal speech aid. Facial scanner records were taken in both smiling and resting positions [41].

3. In 2022, Sun et al. designed a fully digital facial prosthesis for a 13-year-old girl who had lost one eye and parts of the surrounding tissues due to advanced cancer. Digital facial impressions were taken using a portable facial scanner (SCANIFY, Fuel3D Technologies, Ltd., United Kingdom) [42].

4. Silva et al. introduced a fully digital method for creating nasal prostheses in 2022. In this study, molding was done with a facial scanner, and the fabrication process was done with 3D printers [10].

5. In 2024, Jablonski et al. described the steps of designing a nasal prostheses using a facial scanner (Artec Space Spider; Artec 3D) with the meshlab database [43].

### 1. 3. CBCT

The use of 3D imaging instead of conventional molding methods is the future of dentistry. One tool for producing 3D images is Cone Beam Computed Tomography (CBCT). The applications of this imaging in the field of prosthetics include: implant prosthetics, imaging of the temporomandibular joint, maxillofacial prosthetics, diagnosis and evaluation of craniofacial problems, and finally, evaluation and causation of airway problems [13]. The main use of CBCT is in the construction of obturators after maxillectomy for oncological reasons [13][14]. In the following, we will briefly review the articles that suggested the use of CT and CBCT.

1. In 2024, Calderon et al. suggested using high-resolution CT and 3D printers as a fast and affordable method to replace damaged palatal tissues in cancer patients. Although they noted the need to compare this method with conventional methods in other studies [14].

2. In 2019, Tasopoulos created a 3D-printed interim obturator prosthesis using a digital method. In this case, CT was used for impression making [44].

3. In 2021, Ye et al. proposed a fully digital method for constructing an obturator. In this study, CT was taken of the patient’s lesion and a virtual cast was created using the scan file with intraoral scanners and software (Geomagic studio 2012; 3D Systems) [32].

### 2. Classification of Digital Molding by Maxillofacial Defect

In this section, the use of digital molding in various maxillofacial prostheses is examined separately. This classification is as follows (Figure-2).

### 2.1. Lip and Palate Cleft

Patel et al. (2019) compared the accuracy of conventional alginate impressions and digital impressions using a Trios scanner for a 3-month-old patient with a bilateral cleft palate. Both methods demonstrated clinically acceptable accuracy [26].

ElNaghy et al. (2022) compared the accuracy of intraoral scans in patients with unilateral lip and palate cleft. Scans with a Trios 3-Shape scanner were compared to laboratory scans of conventional impressions. The study showed high accuracy for digital impressions in these patients, with errors between 0.01 and 0.1 millimeters [27].

Okazaki et al. (2023) also compared digital and conventional impressions in unilateral lip and palate cleft patients, using a Trios 3-Shape scanner. The study found that the scanned file slightly underestimated the depth of the cleft compared to the conventional impressions, but due to the ease of use for the patient and the absence of aspiration during digital molding, and the lack of significant differences between the two methods, the digital method was recommended [28]. Soliman et al. (2023) evaluated the accuracy of the Medit i700 intraoral scanner in molding 7 infants aged 0-28 days with cleft lip and palate. The study, based on comparable results between the conventional and digital methods, suggested the use of intraoral scanners [45].

Olmos et al. (2023) compared the accuracy of facial and intraoral scanners in recording the nasoalveolar region in patients with unilateral lip and palatal cleft. The study found that intraoral scanners (Trios4:3shape) provided higher accuracy than Canfield facial scanners (Vectra H2) [46].

Villarreal-Martínez et al. (2024) investigated digital nasoalveolar impressions in toddlers with palatal and lip cleft. In this study, three children were examined. In the first case, conventional impression with alginate was done. In the second case, a digital impression was done with a Trios3 scanner, and appliance was made in a resin cast with the help of a 3D printer (250 mW laser, Form2, Formlabs). In the last case, digital impression was done with a Trios3 scanner and the appliance was designed with exocad (ExoCAD GMBH, Align Technology, California, United States). Finally, the appliance was digitally printed. This study indicated that the use of digital methods(group3) is successful and suggested it as an effective method [30].

Gong et al. (2020) introduced a fully digital method for nasoalveolar molding treatment in infants with lip and palatal cleft. A digital impression were taken using a TRIOS scanner. The STL file was transferred to Geomagic Design X 2016 software for segmenting. The appliance was then designed using Rhino software and 3D printed using a bio-compatible material. While this method was fully digital [29], the material used was not recommended for long-term use due to toxicity [47].

### 2. 2. Maxillectomy

Obturators can be divided into two general categories: Interim and definitive. The most important step for making a definitive obturator is impression, while for making interim types, usually not much accuracy is considered. Today, with the introduction of digital methods, the design and manufacturing of interim obturator can be done with high accuracy and speed. In 2019, Tasopoulos made a digital 3D-printed interim obturator prosthesis. In this case, with the help of a printer (Form 2; Formlabs, Inc), a precise cast of the patient was prepared after preparing a CT. An interim obturator was made in a resin cast with the help of silicone denture soft lining [44].

Brucoli’s study in 2020 investigated the use of scanners in making the maxillary obturator. In this study, obturator prostheses were made for 28 patients 5-6 months after surgery, and the quality of the prostheses in terms of speech improvement, lack of leakage, swallowing improvement, and patient satisfaction were investigated. In this study, the scanner (TRIOS; 3Shape) was used for impression. Then, a resin cast was printed and an obturator was made. Almost all patients reported the absence of leakage, and improvement in speech and swallowing was seen in most patients [8].

In the study of Islam in 2023, using a combined digital and conventional method for a defect caused by anterior maxillectomy, an obturator with a metal frame was made. The final obturator in this study had good accuracy [31].

### 2. 3. Mandibulectomy

A major challenge in patients who have undergone mandibulectomy and radiotherapy is trismus, or limited mouth opening. A 2023 study by Gao et al. examined the accuracy of intraoral scans in normal conditions (maximum opening=40 mm) and in cases of trismus (maximum opening 10, 20, and 30 mm). The study showed acceptable results for digital molding in cases of mild and moderate trismus [33].

### 2. 4. Soft Palate Defects

In 2024, Park et al. designed a fully digital, implant-supported overdenture with a pharyngeal speech aid. A gnathometer (Ivoclar AG) tray was used to record the basic cast and intermaxillary relations. The soft palate defect was recorded functionally using a tissue conditioner for 20 minutes. A digital facebow transfer (Zebris for Ceramill; Amann Girrbach AG) was then used, and the records along with facial scans of the patient in rest and smiling positions were sent to the laboratory. The denture was designed in the laboratory and milled using the Ivotion Denture System (Ivoclar AG) [41].

### 2. 5. Ear Prosthetics

Gadallah’s 2023 study compared the accuracy of three intraoral scanners in ear molding. The Primescan, Medit i700, and Panda P2 scanners were used. The study showed significantly higher accuracy for the Medit and Primescan [34].

### 2. 6. Nasal Prosthetics

In 2022, Sun et al. designed a fully digital facial prosthesis for a 13-year-old girl who had lost one eye and surrounding tissues due to cancer. Digital facial impressions were taken using a portable scanner (SCANIFY, Fuel3D Technologies, Ltd., United Kingdom). The missing tissue was designed with the mirror image by the software (Zbrush, Pixologic, Inc., United States). The ocular prosthesis was created separately and added to the facial prosthesis design. The final prosthesis was 3D printed using SLA and silicone [42].

In 2023, Jablonski . compared conventional and digital methods for creating maxillofacial prostheses in 30 participants with nasal and ocular defects. For the digital method, a facial scan was taken using a facial scanner (Artec Space Spider; Artec 3D/ structured light scanner). A wax model was created using a 3D printer (Form 3; Formlabs), and after adjustments, it was printed in silicone. This study showed acceptable results in the digital method. However, it represented more studies necessary [48].

Palousek et al. (2013) used an ATOS facial scanner to create a nasal prosthesis. The design was finalized based on the patient’s age, facial shape, gender, and previous photos. The final prosthesis was 3D printed in wax (ZPrinter 310 Plus; Z Corporation, Burlington, MA, USA). Finally, the definitive prosthesis was made after a try the wax model [49].

### 2. 7. Ocular Prosthetics

In 2013, Ciocca et al. used MRI and a NeXT Engine laser scanner to create an ocular prosthesis. The MRI was used to better capture soft tissues. The prosthesis was created using a mirror image of the contralateral eye. Attachments for glasses were incorporated, and the eyelid and surrounding areas were created using silicone printing [50]. Ciocca et al. in other articles, made nose and ear prostheses with the help of NeXT Engine laser scanning in a similar way [51][52].

## Conclusion

Digital impression techniques have emerged as valuable tools in maxillofacial prosthetics. Intraoral scanners, facial scanners, and CBCT offer various advantages for different types of defects. Intraoral scanners excel in capturing impressions for naso-alveolar, obturator, cleft palate, and ear prostheses. Facial scanners are particularly effective for midface defects, aiding in the creation of occular and nose prostheses. CBCT is primarily used for palate impression and obturator design and fabrication, often in combination with intraoral scanners for optimal results.

Clinical studies have demonstrated the accuracy and patient preference for digital impression methods in maxillofacial prosthetics. Intraoral scanners are favored for naso-alveolar prostheses, while both intraoral scanners and CBCT have shown acceptable accuracy for obturators. CBCT is particularly beneficial for temporary obturator fabrication during surgery. Intraoral scanners are superior to other methods for mandibular impression, even in cases of mild to moderate trismus. Both facial and intraoral scanners can be used for ear prostheses, and facial scanners are helpful for nasal prostheses. MRI plays a crucial role in capturing accurate soft tissue impressions for eye prostheses.

### Clinical advice

Digital impression techniques have the potential to revolutionize maxillofacial prosthetics. Despite their significance, digital methods have been less explored in this field due to their novelty, limited patient base, and the complexity of maxillofacial prosthetics.

Based on the review, clinicians are advised to:

Prioritize intraoral scanners for intraoral defects. These scanners offer superior accuracy, efficiency, and ease of use for both clinicians and patients.

Consider intraoral scanners for nose and ear defects. While not as accurate as facial scanners, intraoral scanners can be used as an alternative when facial scanners are unavailable. However, they are less suitable for eye defects.

Utilize facial scanners for midface areas. Facial scanners provide the most accurate impressions for the nose and can also be used for ear and eye areas.

Exercise caution with mobile phone-based facial scanners. These portable and affordable scanners may have limitations in accuracy, and their use should be complemented by additional methods.

Avoid relying solely on CBCT for interim obturators. While CBCT has been suggested in some studies, it lacks sufficient accuracy. Combining intraoral scanners with CBCT can provide a more reliable approach.

By adopting these recommendations, clinicians can harness the benefits of digital impression technology to improve the accuracy, efficiency, and patient experience in maxillofacial prosthetics.

## Conflict of Interest

None declard.
